# AI-based skin cancer detection: the balance between access and overutilization

**DOI:** 10.1038/s41746-023-00900-0

**Published:** 2023-08-15

**Authors:** Kaushik P. Venkatesh, Marium Raza, Joseph Kvedar

**Affiliations:** Harvard Medical School, Boston, MA USA

**Keywords:** Health care economics, Health services, Health policy, Diagnosis

## Abstract

Gregoor et al. evaluated the healthcare implications and costs of an AI-enabled mobile health app for skin cancer detection, involving 18,960 beneficiaries of a Netherlands insurer. They report a 32% increase in claims for premalignant and malignant skin lesions among app users, largely attributed to benign skin lesions and leading to higher annual costs for app users (€64.97) compared to controls (€43.09). Cost-effectiveness analysis showed a comparable cost to dermatologist-based diagnosis alone. This editorial emphasizes the balance in AI-based dermatology between increased access and increased false positives resulting in overutilization. We suggest refining the diagnostic schemas with new referral pathways to capitalize on potential savings. We also discuss the importance of econometric analysis to evaluate the adoption of new technologies, as well as adapting payment models to mitigate the risk of overutilization inherent in AI-based diagnostics such as skin cancer detection.

The use of AI for medical diagnosis has found an early home at scale in skin cancer. The complex process of diagnosis can involve integrating data on a patient’s symptoms and history, physical exam, lab values, and imaging studies. AI tools, including machine learning and deep learning algorithms, can learn from and efficiently process large volumes of data. Researchers have used AI-based tools to aid in the process of diagnosis in various different contexts, including the detection of diseases of the skin^1^, liver^2^, heart^3^, and other organs^4^. Moreover, other tools are made to interface directly with patients and influence their care.

Gregoor et al. analyze data from the pilot of a mobile health app that used an AI algorithm for skin cancer detection^5^. In 2019, a Dutch health insurance company offered 2.2 million adults free access to this app. Gregoor et al. matched 18,960 users who completed at least one successful assessment with the app to controls who did not use the app. They reported a 32% increase in claims for premalignant and malignant skin lesions among app users compared to non-app users. App users had twice as many biopsies and excisions matched as well as four times (5.9%) the claims for benign skin tumors and nevi compared with controls (1.7%). App users also had fewer claims for malignant skin lesions than controls. The increased benign claims and fewer malignant claims resulted in higher total annual costs for app users (€64.97) vs. controls (€43.09); costs per individual claim for malignant lesions were also higher for app users (€613.36) vs. controls (€520.05). Estimates on the cost of capturing one additional premalignant and malignant skin lesions via the app ranged from €2657 to €488.

## Comparing AI-based skin cancer diagnosis with conventional dermatology

This study is noteworthy in its effort to characterize the healthcare implications of dermatology AI-based diagnosis beyond the existing literature on diagnostic accuracy alone—i.e., cost, cost-effectiveness, and utilization. The reasons for overutilization enabled by this app are manyfold. The app performed as expected from previous reports by the developers—sensitivity of 87–95% and specificity of 70–78%^6,7^. The deployment of this AI skin app at a broad scale shows the real-world costs of more false positives (benign lesion claims) and fewer true positives (malignant lesion claims) compared to the management of non-app users. More false positives and fewer true positives compared with conventional care can take an emotional and financial toll on patients and the healthcare system.

The overall cost-effectiveness of the screening may be comparable to that of a dermatologist. A recent study in the US found that the cost of detecting an additional skin premalignancy or malignancy through total body exams was $2346^8^. Depending on the assumptions of these calculations, the skin app performed at a comparable cost per new positive identification. In context, increased total costs per app user at a comparable cost-benefit ratio suggests that the app users are enjoying more of the “benefits”—i.e., they had more skin lesions diagnosed than non-app users, likely due to increased access. This supports using AI skin apps insofar as access is the limiting determinant of diagnosis.

In addition, the algorithm was preset to flag premalignancies like actinic keratosis, a dysplastic but inherently benign lesion, as high risk. Actinic keratosis is a benign lesion on the same spectrum as squamous cell carcinoma, but with a low risk (0.1%) of malignant transformation^9^. Higher scrutiny in flagging these findings for review/claims should be utilized to triage for more cost-effective care. This may require more nuanced classification schemas as high risk, low risk, or moderate risk. Moderate-risk categories could then be evaluated and triaged by dermatologists and advanced practice providers, with lower reimbursement or diagnostic priority to save costs.

## Addressing overutilization

These data fall along similar findings across other areas of digital health, with the benefit of increased access and timeliness balanced by the risk of overutilization. For example, unique aspects of telehealth, such as more efficient triage and decreased emergency department utilization, suggest a potential for cost-effectiveness, paralleling the benefits and risks observed in dermatology AI. However, in another area of digital health, telehealth implementation in primary care during COVID-19 may have led to overutilization, with some studies suggesting that telehealth visits were used as additions rather than substitutes to in-person visits^10^. A more direct parallel to skin AI may be radiology AI, wherein increased imaging given ease of AI-enabled use led to increased invasive testing and follow-up due to false positives^11^.

As AI implementation will inevitably continue to increase, a few different strategies will be necessary to address the conundrum of overutilization. Primarily, the use of AI algorithms should be rationalized before implementation. When appropriate, AI algorithms should be evaluated against traditional methods using econometric analysis and pilot studies such as that of Gregoor et al. The rate and cost of true positive identification should be assessed against the rate of false positives and the performance of traditional care. On a systems level, this will require regulators and administrators to establish guidelines for the responsible use of AI diagnostics. The V3 framework for biometric monitoring can be adapted for AI diagnostics more broadly; it involves verification of the technology with preset criteria, analytical validation of the algorithm, and clinical validation in a real-world target context^12^.

Payment and incentive models will need to be adapted as well. The current per-use reimbursement models, while feasible for early AI products, may result in the overuse of AI, analogous to experiences with traditional medical devices. In the pursuit of value-based care, reimbursement should incorporate outcomes instead of volume. In addition, when piloting new AI devices like the skin cancer app in this study, payers should utilize advanced market commitments and time-limited reimbursements for new AI applications. Such an approach can more sensitively control the adoption of AI technologies and mitigate risks of overuse^13^.

As AI for clinical diagnostics moves more broadly into the implementation stage, the threat of overutilization should be anticipated. Rising to this challenge will require adjustment to payment models and evidence-based stewardship.
